# School-Based Preventive Dental Program in Rural Communities of the Republic of Armenia

**DOI:** 10.3389/fpubh.2019.00243

**Published:** 2019-08-28

**Authors:** Hamlet Gasoyan, Armen Safaryan, Lusine Sahakyan, Nairuhi Gasoyan, William E. Aaronson, Robert A. Bagramian

**Affiliations:** ^1^Department of Health Services Administration and Policy, College of Public Health, Temple University, Philadelphia, PA, United States; ^2^Visiting Scientist in Health Services Administration and Policy, Yerevan State Medical University, Yerevan, Armenia; ^3^Faculty of Public Health, Yerevan State Medical University, Yerevan, Armenia; ^4^Children of Armenia Fund, Yerevan, Armenia; ^5^School of Dentistry, University of Michigan, Ann Arbor, MI, United States; ^6^Dean Emeritus, School of Public Health, American University of Armenia, Yerevan, Armenia

**Keywords:** caries prevention, primary schoolchildren, fluoride toothpaste, school-based intervention, Armenia

## Abstract

**Objectives:** This paper describes a school-based preventive dental program implemented in 14 rural schools within nine villages of Armenia. As part of the program, school-based toothbrushing stations (called Brushadromes) were installed in the participating schools. The intervention included school-based supervised toothbrushing with fluoride toothpaste and oral hygiene education.

**Methods:** The study evaluates the prevalence and levels of dental caries among rural schoolchildren in 2013 (before the implementation of the preventive program, referred to as a pre-intervention group) and 2017 (4 years after the start of the program, referred to as an intervention group) in two randomly selected villages where the program was implemented. A repeated cross-sectional study design was used. The prevalence of caries and the number of decayed, missing, and filled teeth in permanent dentition (DMFT) and primary dentition (dmft) were recorded among 6–7 and 10–11-year-old schoolchildren in 2013 (*n* = 166) and 2017 (*n* = 148). The pre-intervention and intervention groups include different children in the same age range, from the same villages, examined at different time points. In both instances, they represented over 95% of the 6–7 and 10–11-year-old student populations of the studied villages. Pearson Chi-square, Fisher's Exact test, independent *t*-test, and quasi-likelihood Poisson regression were utilized for data analysis.

**Results:** Schoolchildren involved in the intervention had significantly less decay levels compared to same-age pre-intervention groups. For 10–11-year-old schoolchildren involved in the program, the mean number of permanent teeth with caries was lower by a factor of 0.689 (lower by 31.1%), *p* = 0.008, 95% CI, 0.523; 0.902, compared to the 10–11-year-old pre-intervention group, after controlling for age, sex, child's socio-economic vulnerability status, the village of residence, and the number of permanent teeth with fillings.

**Conclusions:** The study indicates a significantly lower level of caries among schoolchildren in the studied two villages where the intervention was implemented. The described intervention is particularly suitable in rural settings where water fluoridation is not available and homes have limited availability of running water.

## Introduction

Dental caries remains one of the most prevalent chronic conditions among children in many countries (1, 2). The reported outcomes of dental caries and poor oral health in children are well-documented and range from serious health problems, such as dental abscess, to negative effects on nutrition, growth, and development, as well as children's school performance (3, 4). In the post-Soviet Republics, including Armenia, there has been very little data collected on the prevalence and levels of dental caries. A 2005 study conducted by the American University of Armenia reported an 86% prevalence of dental caries in a sample of 12 year-old schoolchildren in one of the provinces of Armenia (5).

Armenia is classified by the World Bank in the upper-middle-income economies tier (6). After its independence in 1991, the Armenian healthcare system experienced decentralization and partial privatization, leading to increased out-of-pocket payments and limited access for the poorest households to essential health and dental services (7, 8). Armenia is subdivided into 11 administrative divisions (10 provinces and the capital—Yerevan). Poverty and underdeveloped infrastructures are typical for the country's remote villages (9, 10). The percentage of people in the country living below the state-defined poverty level was 25.7% in 2017 (9).

Like in many middle-income countries, water fluoridation, or other similar mass-preventive methods, is not provided in Armenia (11). Inadequate knowledge on dental hygiene among children and their parents, unhealthy nutritional habits, and limited availability of running water in many homes of remote villages prompted the Children of Armenia Fund (COAF) to implement a school-based supervised toothbrushing intervention with fluoride toothpaste and oral hygiene education project in three provinces of Armenia.

There have been previous studies documenting the efficacy of school-based supervised toothbrushing programs in England, Scotland, and Australia (12–14). The authors are not aware of any similar studies that were conducted in the context of post-Soviet countries.

This paper presents a school-based preventive dental program implemented in nine villages within three provinces of Armenia. In addition, the paper evaluates the prevalence and levels of dental caries among rural schoolchildren ages 6–7 and 10–11 in 2013 (before the implementation of the preventive program, referred as a pre-intervention group) and 2017 (4 years after the start of the program—intervention group) in program implementation areas.

## Methods

### Intervention

Within the preventive dental program, in 2013, COAF installed school-based toothbrushing stations (called Brushadromes) in 14 rural schools of nine (Karakert, Arteni, Dalarik, Lernagog, Shenik, Miasnikian, Bagaran, Yervandashat, Argina) villages within three (Armavir, Aragatsotn and Lori) provinces of Armenia. The “Brushadrome” is a room, next to the cafeteria, equipped with multiple sinks and individual cabinets for dental hygiene supplies which allow schoolchildren to brush their teeth after lunch. The program began in 2013 and is currently running with an expansion to 22 more villages.

The intervention was 5 days per week (after lunch) of supervised toothbrushing, using fluoridated toothpaste and a medium soft brush (products by Colgate®) at school, coupled with oral hygiene education for children and parents. The school-based supervised toothbrushing was conducted for overall 135 school days per year (excluding cold months of the year, when the school cafeteria was closed, and out-of-school days). The toothbrush was replaced after ~70 days of use, at the beginning of the Fall and Spring semesters, as well as whenever there was noticeable toothbrush wear. The fluoride concentration in the toothpaste was 1,000 p.p.m.; no other relevant ingredients were included in the toothpaste. Children also received oral hygiene products for home use.

Children were instructed on proper oral hygiene and brushing techniques, as well as supervised while brushing, either by the school nurse or primary school teachers; both received the same training as part of the intervention. The Vertical Sweeping Brushing Technique was followed. The brushing time was set to 2 min and timed via sand timers. Parents of the schoolchildren were also educated on oral hygiene topics and encouraged to monitor whether the child brushed the teeth twice a day at home.

The utilization of the “Brushadromes” by the schoolchildren was very high throughout the intervention.

### Study Design

This study employed a repeated cross-sectional design. The prevalence of caries and the number of decayed, missing and filled teeth in permanent dentition (DMFT) and primary dentition (dmft) were recorded among 6–7 and 10–11-year-old schoolchildren at two time-points: in 2013 (before the initiation of the intervention) and in 2017 (four years after the start of the intervention).

### Setting and Data Sources

In 2013, COAF examined 6–7 and 10–11-year-old schoolchildren residing in nine participating villages, as part of its community needs assessment. The 2017 examination targeted 6–7 and 10–11-year-old schoolchildren residing in two of the randomly selected villages (Karakert and Lernagog) from the 2013 study. The latter was done due to time and resource limitations. Many of the homes in both villages do not have running water.

All examinations were by visual assessments only; only mouth mirrors were used with natural light. The children were in an upright position during the examinations. The tooth was considered decayed if there was an untreated or secondary caries at least into dentine. No primary incisor was recorded as missing to reduce error due to the physiological loss in the age group 6–7.

The 2013 examination was carried out by a medical doctor trained in oral health (LS). The 2017 examination was conducted by a general practice dentist (AS). To assure the reliability and validity of the data, the examiner who completed the 2013 screening participated in a calibration meeting with the examiner who conducted the 2017 round of the examinations before its initiation. The first examiner was also available for consultations to the second examiner throughout the second round of examinations.

According to statements from COAF, school officials, and parents, during 2013–2017 no other dental mass-preventive program or water fluoridation was available for the discussed population. Permission to conduct the study was obtained from the Research Ethics Committee at Yerevan State Medical University and the research has been conducted in full accordance with the World Medical Association Declaration of Helsinki. Each parent or guardian received a study information sheet and provided written consent for their child to participate. The consent procedure was approved by the Research Ethics Committee at Yerevan State Medical University and was carried out following the local law.

### Study Size and Participants

The decay prevalence and levels were calculated for the primary teeth at the age group 6–7 and for the permanent teeth at the age group 10–11 among schoolchildren residing in the selected two villages at two-time points: pre-intervention (*n* = 166) and 4 years after the start of the intervention (*n* = 148). In both rounds of the examinations, over 95% of the targeted-aged schoolchildren of the selected two villages participated in the examinations. The selected age groups correspond to the lower and upper age bounds of the primary schoolchildren population in Armenia. Schoolchildren ages 8–9 were not included in this study due to the challenges introduced by their mixed dentition. We also present the 2013 caries prevalence data covering all nine villages that were included in the initial community needs assessment (*n* = 422).

To be included in the pre-intervention group, schoolchildren had to be 6–7 or 10–11 years old as well as be residing in the selected two villages and attending one of the three local schools. 2017 (intervention group) examination had the same eligibility criteria. In addition, it required that schoolchildren participate in the intervention for at least 1 year for the age group 6–7 and 3 years for the age group 10–11.

### Variables

The key outcome variables within this study include caries prevalence and levels as well as DMFT/dmft indices. Caries levels were defined by the number of decayed teeth (components D and d in the respective indices). For the calculation of prevalence, at least one untreated decayed tooth was considered as a threshold.

The primary predictor variable in the multivariable regression model is participation in the intervention. Covariates include participant's age, sex, socio-economic vulnerability status, the village of residence, and the number of teeth with fillings. The socio-economic vulnerability status was assigned to either vulnerable or not-vulnerable categories, based on the records from COAF's database of vulnerable children. The latter is maintained by community social workers and is determined based on a 22-item checklist. The number of permanent teeth with fillings was included in the multivariable model as a proxy for access to dental services. The village of residence was included to account for village-level unobservable characteristics.

### Statistical Analysis

The statistical significance of differences in decay prevalence was tested by using Pearson Chi-square and Fisher's Exact test (based on the count of decay-free cases) and 95% Confidence Intervals were calculated using the modified Wald method. Differences in mean decay levels, as well as DMFT and dmft scores, were tested using independent *t*-tests.

Poisson distributions are often used in modeling count data (15). A quasi-likelihood Poisson regression was performed to investigate the association of participation in the intervention, age, sex, socio-economic vulnerability status, the village of residence, the number of permanent teeth with fillings, and the levels of permanent caries among the 10–11-year-old children from the two villages. Significance level was determined using an alpha of 0.05. The analyses were performed with IBM SPSS Statistics for Windows, version 24.0., Armonk, NY: IBM Corp. and R (R statistics), version 3.5.1.

## Results

### Sample Characteristics

The pre-intervention group in the selected two villages included 80 children in the 6–7-year-old age group (53% male, 47% female) and 86 in the 10–11-year-old age group (59% male, 41% female). Approximately 57% of the pre-intervention group was from Karakert and 43% from Lernagog village.

The intervention group in the selected two villages included 73 participants in the 6–7-year-old age group (57% male, 43% female) and 75 in the 10–11-year-old age group (51% male, 49% female). This group included 64% of its participants from Karakert and 36% from Lernagog village.

While the participation in the intervention was voluntary, our consultations with the program administrators, schoolteachers, and nurses as well as the self-reported data by schoolchildren and their parents indicated almost no refusals to participate in the intervention.

### Prevalence of Decay

The 2013 examination involving the selected two villages showed that 98.75% (95% CI, 92.59; 99.99) of the children aged 6–7 had decay in primary dentition and 82.56% (95% CI, 73.08; 89.25) of the children aged 10–11 in permanent dentition. The 2013 prevalence data pooled from all nine villages shows almost identical baseline numbers of decay prevalence (Table 1). The 2017 examination among the two villages revealed 91.27% (95% CI, 82.89, 96.49) prevalence in the age group 6–7 and 73.33% (95% CI, 62.31, 82.09) in the age group 10–11. There was a larger difference in the prevalence of caries in the age group 10–11 (9.23%) than in the 6–7 group (7.48%). This coincides with the exposure to the intervention. However, the difference in prevalence in the 2013 and 2017 examinations in the two villages was not statistically significant.

**Table 1:** Prevalence of caries pre-intervention and 4 years after the start of the program.

**Group**	**Pre-intervention group prevalence of decay in % [95% CI] (*****n*****; mean age)**		**Intervention group prevalence of decay in % [95% CI] (*n*; mean age)**
	All nine villages, 2013 data	Karakert and Lernagog villages only, 2013 data	Karakert and Lernagog villages only, 2017 data
6–7-year-old (primary teeth)	97.36% [94.22; 98.92] (*n* = 227; 6.67)	98.75% [92.59; 99.99] (*n* = 80; 6.59)	91.27% [82.89; 96.49] (*n* = 73; 6.95)
10–11-year-old (permanent teeth)	81.33% [74.30; 86.81] (*n* = 195; 10.12)	82.56% [73.08; 89.25] (*n* = 86; 10.07)	73.33% [62.31; 82.09] (*n* = 75; 10.40)

^*^p < 0.05; ^**^p < 0.005.

### Levels of Decay

The mean number of decayed primary teeth among the intervention group aged 6–7 was significantly lower (−1.57, *p* < 0.05) compared to the same age pre-intervention group (Table 2). The mean number of decayed permanent teeth among the intervention group aged 10–11 was also significantly lower (–0.61, *p* < 0.05) compared to the same-aged pre-intervention group (Table 3).

**Table 2:** Decay levels in primary teeth pre-intervention and four years after the start of the program among 6–7-year old schoolchildren in Karakert and Lernagog villages.

**Assessment**	**Pre-intervention group Mean (*SD*)**	**Intervention group Mean (*SD*)**	**Mean difference pre-intervention group – intervention group, [95% CI]**
d (decay in primary teeth)	7.80 (3.43)	6.23 (4.12)	−1.57^*^ [−2.78; −0.35]
dmft	8.24 (3.50)	7.29 (4.30)	−0.95 [0.64;−2.21]
f (number of primary teeth with fillings)	0.08 (0.47)	0.30 (0.76)	0.23^*^ [0.10; 0.02]

* p < 0.05;

^**^p < 0.005.

**Table 3:** Decay levels in permanent teeth pre-intervention and four years after the start of the program among 10–11-year old schoolchildren in Karakert and Lernagog villages.

**Assessment**	**Pre-intervention group Mean (*SD*)**	**Intervention group Mean (*SD*)**	**Mean difference pre-intervention group – intervention group, [95% CI]**
D (decay in permanent teeth)	2.27 (1.59)	1.65 (1.48)	−0.61^*^ [−1.09; −0.14]
DMFT	2.50 (1.73)	1.76 (1.53)	−0.74^**^ [−1.25; −0.23]
F (number of permanent teeth with fillings)	0.09 (0.36)	0.08 (0.32)	−0.01 [−0.12; 0.09]

* p < 0.05;

** *p < 0.005*.

Tables 2, 3 also present the mean values and differences in DMFT and dmft indices in the intervention vs. pre-intervention groups. The mean number of primary teeth with fillings among the intervention group aged 6–7 was slightly higher (0.23, *p* <0.05) compared to the same-aged pre-intervention group. However, across the board, components D/d (decay) remained very high and constituted the largest portion of the DMFT and dmft indices. For example, in the pre-intervention group aged 6–7, the mean number of decayed primary teeth was 7.80 and in the ages 10–11, the mean number of decayed permanent teeth was 2.27. As opposed to that, the mean values of components F/f (fillings) were very low across the board (<0.50).

### Results of the Multivariable Model

A slight overdispersion was detected in the data obtained from the 10–11-year-old pre-intervention and intervention groups (dispersion parameter = 1.22), indicating that there was somewhat greater variability in the data than would be expected based on the Poisson model. To control for this, a quasi-likelihood Poisson model was used.

According to the multivariable regression model results, the mean number of permanent teeth with caries in the 10–11-year-old intervention group was lower by a factor of 0.689 (lower by 31.1%), *p* = 0.008, 95% CI, [0.523; 0.902] compared to the 10–11-year-old pre-intervention group, after controlling for age, sex, child's socio-economic vulnerability status, the village of residence, and the number of permanent teeth with fillings (Table 4). The individual estimates of other covariates in the model should not be interpreted in the same way as the primary predictor (16).

**Table 4:** Association of participation in the intervention, age, sex, socio-economic vulnerability status, village of residence, the number of permanent teeth with fillings, and the number of permanent teeth with caries, among the 10–11-year-old children from the two villages.

**Parameter**	**Exponentiated poisson regression coefficient**	**95% CI (exponentiated)**	***p*-value**
Intervention group (reference category non-intervention group)	0.689	[0.523; 0.902]	0.008
Female (reference category male)*^¶^*	1.034	[0.806; 1.324]	0.790
Socio-economic status vulnerable (reference category non-vulnerable)*^¶^*	1.034	[0.637; 1.593]	0.886
Village Lernagog (reference category Karakert)*^¶^*	0.917	[0.703; 1.191]	0.519
Age (one-year increase)*^¶^*	1.132	[0.832; 1.512]	0.418
Number of permanent teeth with fillings (one-unit increase)*^¶^*	0.787	[0.498; 1.150]	0.261

Dependent variable: Number of permanent teeth with caries;

¶ *Covariates in the model, these estimates should not be interpreted in the same way as the primary predictor*.

## Discussion

This study indicates a high prevalence of dental caries among rural children, ages 6–7 and 10–11, in nine villages of Armenia. Results also showed that access to dental restorative services remains very low among the studied schoolchildren population in the two villages. These findings indicate the need for further dialogue on the implementation of oral health preventive measures in the remote rural communities.

The study results indicate that those involved in the intervention had significantly less decay levels in their primary dentition after 1 year and in the permanent dentition after 3-year exposure, compared to same-age schoolchildren examined before the initiation of the program. The findings of this study can be placed into context with those conducted in other countries. For example, a similar intervention among 5–6-year-old schoolchildren in England, including once-a-day, at school, during term time, teacher-supervised toothbrushing with commercial toothpaste, showed that children in the intervention group had an overall 10.9% lower mean total caries increment (2.60 vs. 2.92, *p* < 0.001) compared to those in the non-intervention group (12). Another study including supervised toothbrushing with a fluoridated toothpaste in high-caries-risk children living in deprived areas of Tayside, Scotland, showed that children in the intervention group had a 32% lower D_1_ level (all visible cavitated and non-cavitated lesions in enamel and dentine) 2-year mean caries increment on first permanent molars compared to the control group (13).

Toothbrushing with a fluoridated toothpaste is an effective means of reducing caries and periodontal disease and those who practice good oral hygiene at an early age are more likely to maintain it throughout their lives (17, 18). However, in many low-income families in the rural villages of Armenia twice-daily toothbrushing is not a usual practice (5).

Some study limitations should be highlighted. First, the intervention did not produce its results under ideal conditions, and the study was unable to account for all “real life” scenarios (e.g., possible changes in diet, etc.). Second, the study cross-sectional design and the absence of a concurrent control group introduces challenges regarding group comparability. Third, both rounds of the examinations were under natural lighting, limiting the ability to detect caries. Finally, data on the prevalence and levels of caries among rural schoolchildren in Armenia are limited, making it difficult to conduct comparisons.

School-based mass preventive programs using supervised toothbrushing with a fluoridated toothpaste could be an effective preventive measure in rural communities of Armenia. Leaders of the organization (COAF) have begun to add additional preventive methods to this program such as topical fluoride treatments. However, due to the timing of the introduction of these components, their impact did not apply to the studied sample.

The COAF has made the preventive dental program a part of its core operations and is utilizing various fundraising mechanisms to sustain it. The formed partnerships with local schools contribute to its low cost of operations.

Further studies could inform whether starting the intervention at earlier ages in kindergarten and adding other low-cost components, such as parental education on oral health, supervised flossing, topical fluoride, and fluoride varnish applications could result in more reduction of the prevalence and levels of caries among children in deprived rural communities of Armenia. It is anticipated that we will see greater reductions in dental caries in this population as children participate for a longer period. The value of this program will need to be evaluated as children are exposed and participate in all the years that they are in school.

## Data Availability

The datasets generated for this study are available on request to the corresponding author.

## Ethics Statement

Permission to conduct the study was obtained from the Research Ethics Committee at Yerevan State Medical University (Armenia) and the research has been conducted in full accordance with the World Medical Association Declaration of Helsinki.

## Author Contributions

HG, AS, LS, WA, and RB: concept and design. HG, AS, LS, and NG: acquisition, analysis, or interpretation of data. HG: drafting of the manuscript. All authors provided critical revisions of the manuscript for important intellectual content, reviewed the manuscript and approved the manuscript for submission, and attest they meet the ICMJE criteria for authorship.

### Conflict of Interest Statement

The authors declare that the research was conducted in the absence of any commercial or financial relationships that could be construed as a potential conflict of interest.
